# Polymorphic Variants of *AGT, ABCA1*, and *CYBA* Genes Influence the Survival of Patients with Coronary Artery Disease: A Prospective Cohort Study

**DOI:** 10.3390/genes13112148

**Published:** 2022-11-18

**Authors:** Anna Balcerzyk-Matić, Tomasz Nowak, Katarzyna Mizia-Stec, Joanna Iwanicka, Tomasz Iwanicki, Paweł Bańka, Alicja Jarosz, Artur Filipecki, Iwona Żak, Jolanta Krauze, Paweł Niemiec

**Affiliations:** 1 Department of Biochemistry and Medical Genetics, School of Health Sciences in Katowice, Medical University of Silesia, Medykow Street 18, 40-752 Katowice, Poland; 2 First Department of Cardiology, School of Medicine in Katowice, Medical University of Silesia, 47 Ziołowa St., 40-635 Katowice, Poland; 3 American Heart of Poland, Armii Krajowej 101 Avenue, 43-316 Bielsko-Biała, Poland

**Keywords:** coronary artery disease, polymorphism, survival

## Abstract

Genetic factors can influence the risk of coronary artery disease (CAD) and the survival of patients. Our previous research led to the identification of genetic variants predisposing to CAD in the Polish population. Since many of them affect the clinical phenotype of the disease, the aim of this study was searching for genetic factors potentially influencing survival in patients with CAD. The study included 276 patients hospitalized due to coronary artery disease. The database of medical history and genotypic results of 29 polymorphisms were used. The endpoint was defined as death from cardiovascular causes. Survival was defined as the period from angiographic confirmation of CAD to death from cardiovascular causes. Three of all the analyzed genes were associated with survival. In the case of the *AGT* (rs699) and *ABCA1* (rs2230806) genes polymorphisms, the risk of death was higher in GG homozygotes compared to the A allele carriers in the 10-year period. In the case of the *CYBA* (rs72811418) gene polymorphism, the effect on mortality was shown in both 5- and 10-year periods. The TA heterozygotes were predisposed to a higher risk of death than the TT homozygotes. Concluding, the *AGT, ABCA1*, and *CYBA* genes polymorphisms influence the risk of death in patients with CAD.

## 1. Introduction

The multifactorial model of inheritance of coronary artery disease (CAD) assumes strong genetic heterogeneity of the atherosclerotic phenotype, which is confirmed by the results of numerous studies, including large-scale genome-wide association studies [1,2]. According to current knowledge, there are inter-population differences in the frequency of genetic variants considered as genetic risk factors for CAD [3,4].

Twenty years ago, our team started research with the primary goal of expanding knowledge about the genetic background of coronary artery disease in the Polish population. We have tested 29 genotypic variants at 13 loci (in functional genes and one of the intergenic region) in a classic case-control model in a group of patients with premature angiographically confirmed CAD (up to 60 years of age). Polymorphisms were selected systematically over the years on the basis of increasing knowledge, initially based on the results of case-control, then genome-wide association studies. Our research led to the finding of genetic risk factors for CAD in the Polish population, as well as the identification of gene-traditional risk factors interactions [5,6,7,8,9,10,11,12,13,14,15,16,17,18,19,20,21,22,23,24]. This made it possible to identify subgroups of patients genetically predisposed to a stronger response to the exposure of common traditional risk factors such as cigarette smoking, lipid disorders, and others.

Since many of the polymorphisms we investigated affect the clinical phenotype of the disease, we decided to check whether their variants modulate the survival of patients with CAD. The current study aimed to evaluate the impact of 29 previously analyzed polymorphisms on the survival of Polish patients with angiographically documented CAD in a 5- and 10-year follow-up.

## 2. Materials and Methods

### 2.1. Study Design and Sample

We performed prospective analysis of the genetic risk factors for cardiovascular death. The study included a cohort of 276 patients hospitalized in the years 2001–2011 due to early CAD. Patients were recruited from the First Department of Cardiology, Upper Silesian Medical Centre in Katowice, Poland, and First Department of Cardiac Surgery, Upper Silesian Medical Centre in Katowice, Poland. The study group included patients with angiographically confirmed CAD with more than 50% diameter stenosis of at least one major coronary vessel. The coronary angiography was performed by Judkin’s method. The group comprised of 31% women and 69% men, aged 25–55 (mean 45.4 ± 6.4). The exclusion criteria were: clinical diagnosis of cardiomyopathy, coagulopathy, collagenosis and acute poisoning (e.g., CO, amphetamine).

For the analysis, a database was used that included the results of molecular analyses of 29 polymorphisms in 12 genes and the 9p21 intergenic region, as well as data from a detailed medical history. The relationships of particular polymorphisms with the risk of disease have been described in previously published articles and summarized in Table 1. The studies we refer to were approved by the ethics committee of the Medical University of Silesia in Katowice (Poland), and informed written consent was obtained from each participant.

### 2.2. Follow-Up and Events

The endpoint in the present study was death from cardiovascular causes, according to the International Statistical Classification of Diseases and Related Health Problems (ICD-10). The complete observation was the occurrence of the endpoint, while the censored observation was the absence of the endpoint (survival) or death due to causes other than cardiovascular disease. Data on the date and causes of death of patients (ICD-10) were obtained from the Katowice City Hall and the Central Statistical Office of Poland (Główny Urząd Statystyczny). Survival was defined as the period (in years) from angiographic confirmation of CAD (which is equivalent to the time of inclusion in the study) until death from cardiovascular causes.

### 2.3. Statistical Methods

Statistical analyses were performed using the Statistica 13.0 software (TIBCO Software Inc, Palo Alto, CA, USA). The analysis of the survival curves was presented using the Kaplan-Meier estimator. The significance of the difference between the survival curves was analyzed using the log-rank test for two trials (allele carrier-state vs. homozygosity) and the χ2 test for multiple trials (comparison of survival curves by genotype). The influence of the assessed parameters on the risk of death was analyzed using the Cox proportional hazard model. For significant parameters, the hazard ratio was presented together with the 95% confidence interval. The differences for which *p* < 0.05 were considered statistically significant. The analysis was performed in a univariate model, then genetic variants showing statistical significance were analyzed in a multivariate model. The results were also adjusted for traditional risk factors, including sex, age, body mass index, type 2 diabetes, hypertension, hyperlipidemia, and smoking.

## 3. Results

### 3.1. Endpoint and Traditional Risk Factors

During the 5-year follow-up, 16 of the patients (5.80%) died, including 14 (5.07%) patients who died of cardiovascular causes (87.50% of deaths). The average survival time of these subjects in the analyzed period was 2.44 (1.75) years. During the 10-year follow-up, 39 (14.13%) patients died, including 32 patients (11.59%) who died of cardiovascular causes (82.05% of deaths). The mean survival time of these subjects in the analyzed period was 5.22 (3.00) years. Cardiovascular causes of death, according to the ICD-10 classification, are presented in Table 2.

Diabetes was the only traditional risk factor that influenced mortality from cardiovascular causes in the five-year follow-up (*p* = 0.02). In the case of a ten-year follow-up, none of the examined factors significantly differentiated patients who died of cardiovascular causes from the remaining patients, although there was a tendency to a higher prevalence of male sex, cigarette smoking, hypertriglyceridemia, critical stenosis and multivessel disease in the group of patients who died (Table 3).

### 3.2. Genetic Factors and Survival

Three of all the analyzed genes were associated with survival in the single factor analysis. These were the *AGT, ABCA1*, and *CYBA* genes.

**Figure 1:**
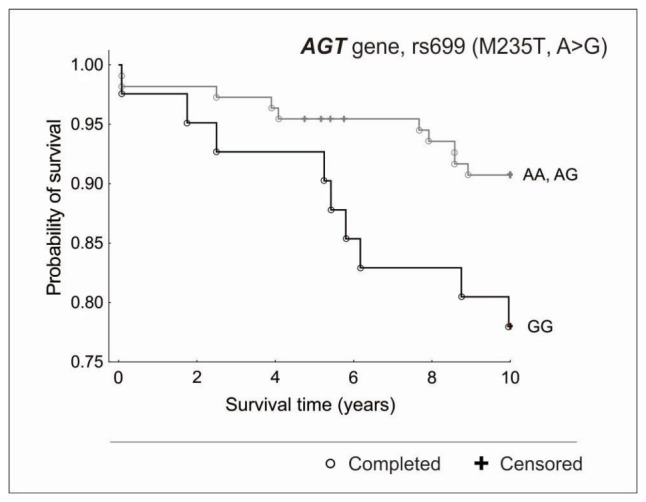
Kaplan-Meier survival curves for CAD patients by *AGT* gene, rs699 (M235T, A > G) in a 10-year observation (*p* = 0.04 in the Cox proportional hazard model, *p* = 0.04 in the log-rank test).

In the case of the *AGT* gene, differences were observed only in the 10-year follow-up, in the recessive model. The risk of death was more than 2.5 times higher for GG homozygotes compared to the allele A carriers (HR = 2.53, 95% CI = 1.03–6.23, *p* = 0.04) (Figure 1).

**Figure 2:**
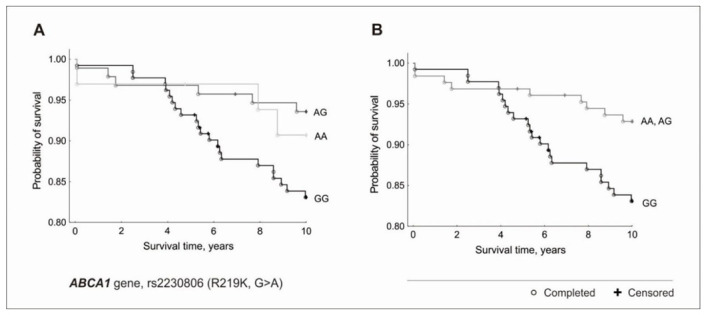
Kaplan-Meier survival curves for CAD patients by *ABCA1* gene (rs2230806 R219K, G > A): (**A**) 10-year survival in additive model (*p* = 0.04 in the Cox proportional hazard model, *p* = 0.06 in the χ2 test) (**B**) 10-year survival in a dominant model (*p* = 0.02 in the Cox proportional hazard model, *p* = 0.02 in the log-rank test).

In the case of the *ABCA1* gene, differences were also observed only in the 10-year follow-up. A significantly higher risk of death was found both in the additive model (genotypes) (HR = 1.91, 95% CI = 0.57–6.38, *p* = 0.04) and in the dominant model, where the risk of death was almost 2.5-times higher for the GG homozygotes compared to the allele A carriers (HR = 2.48, 95% CI = 1.14–5.39, *p* = 0.02) (Figure 2).

**Figure 3:**
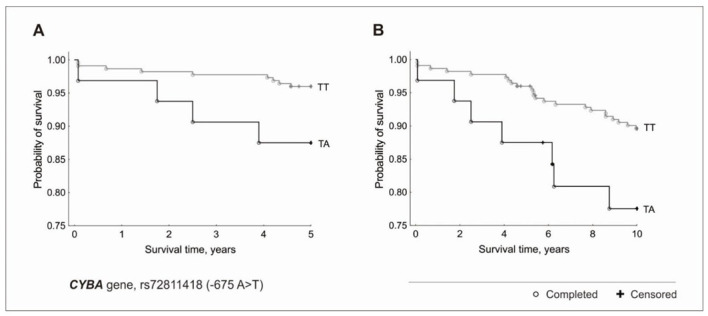
Kaplan-Meier survival curves for CAD patients by *CYBA* gene, rs72811418 (−675 A > T): (**A**) 5-year survival in additive model (*p* = 0.048 in the Cox proportional hazard model, *p* = 0.04 in the χ2 test). (**B**) 10-year survival in additive model (*p* = 0.04 in the Cox proportional hazard model, *p* = 0.04 in the χ2 test).

In the case of the *CYBA* gene, the AT heterozygotes in comparison to the TT homozygotes were characterized by an over three-times higher risk of death over a 5-year period (HR = 3.27, 95% CI = 1.01–10.61, *p* = 0.048) and over a two-times higher risk of death over a 10-year period (HR = 2.37, 95% CI = 1.01–5.53, *p* = 0.04) (Figure 3). No AA homozygotes were observed in the study group.

The multivariate analysis, taking into account all gene variants significant in the univariate analysis, showed an effect on the survival of the *CYBA* gene (for the AT heterozygotes, HR = 3.27, 95% CI = 1.00–10.61, *p* = 0.049) in a 5-year follow-up. In the 10-year observation, significant differences in survival concerned the *AGT* gene (for the GG homozygotes HR = 2.53, 95% CI = 1.03–6.23, *p* = 0.04).

After adjustment for risk factors, including sex, age, body mass index, type 2 diabetes, hypertension, hyperlipidemia, and smoking, a significant effect on the survival was observed only for *CYBA* gene A allele carrier-state in the 10-year follow-up (HR = 2.84, 95% CI = 1.15–7.02, *p* = 0.02).

## 4. Discussion

In the current cohort study of patients with angiographically documented CAD, we analyzed the impact of 29 genetic polymorphisms on survival over 5- and 10-year follow-up. The results of our study suggest that polymorphic variants of the *AGT, ABCA1*, and *CYBA* genes may be responsible for the differentiated survival of patients with CAD. In the 10-year follow-up, the risk of death was higher in GG homozygotes of the polymorphisms: rs699 (A > G) of the *AGT* gene and rs2230806 (G > A) of the *ABCA1* gene. Regarding the 5- and 10-year follow-up, the TA heterozygosity of the *CYBA* gene was a risk factor for death. Multivariate analysis showed that significant differences in survival concern the *CYBA* gene (5-year follow-up) and *AGT* gene (10-year follow-up). After adjustment for traditional risk factors, a significant effect was observed for *CYBA* gene in the 10-year follow-up.

The results of the current work do not seem to be accidental. Alleles and genotypes that in the current study increased the risk of death were associated with CAD (*AGT*) or interacted with traditional risk factors/other SNPs that increase the risk of the disease (*ABCA1, CYBA*) in our previous studies [6,14,20]. The potential impact of these three polymorphisms on CAD and survival is discussed below in the context of the available literature.

Angiotensinogen (AGT) is a precursor of biologically active components of the renin-angiotensin system (RAS). It is encoded by the *AGT* gene, which is highly variable, although most mutations are rare. A common polymorphism in the Caucasian population is rs699 (A > G). It was found that plasma angiotensinogen concentration is about 20% higher in the carriers of the G allele (235T) compared to the AA homozygotes [25]. The G allele and the GG genotype were associated with CAD in Caucasian and Asian populations [26], and heart failure in Caucasian populations [27]. The GG genotype has also been associated with hypertrophic cardiomyopathy in some populations, and especially with sporadic hypertrophic cardiomyopathy [28].

The *ABCA1* gene encodes a protein that acts as a cholesterol removal pump during cellular lipid removal. Mutations in the *ABCA1* gene lead to Tangier disease (lack of plasma HDL, premature CAD), and its polymorphisms may modulate blood lipid levels. One such SNP is the rs2230806 polymorphism (G > A) (R219K polymorphism). The results of meta-analyses indicate that the AA genotype may be associated with higher plasma HDL cholesterol levels [29,30] and carrying the A allele of the rs223086 polymorphism significantly reduces the risk of CAD [29,31,32]. The A allele of the rs2230806 was also associated with a reduced risk of premature heart disease in patients with familial hypercholesterolemia [33].

The *CYBA* gene encodes a membrane protein that is an auxiliary subunit of the cytochrome b558, a component of the complex of NADPH oxidases, proteins responsible for the production of superoxide (O2.-). The *CYBA* gene is polymorphically variable, also within the promoter sequence where the rs72811418 (−675 A > T) SNP is located. Pioneering studies indicate that the T allele is associated with increased NADPH oxidase activity and greater superoxide anion production, while the TT genotype is associated with hypertension and greater thickness of the intima-media complex in the carotid artery wall [34,35]. In subsequent studies, the AA genotype was associated with lower HDL levels and increased triglycerides in patients with non-alcoholic steatohepatitis [36]. The previous study on the Polish population also showed an association between another *CYBA* polymorphism, namely c.214C > T (rs4673), and two end points: all-cause death and elective percutaneous coronary intervention (PCI) and/or coronary artery bypass grafting (CABG). It can be a further confirmation that the *CYBA* gene can influence the course of CAD [37].

Our findings may not only help to explain differences in survival between individual patients, but also indicate potential targets for further cellular and molecular research, which can improve our understanding of the course and progression of atherosclerotic changes. Novel strategies, including gene-editing, which are used to mimic target SNPs may confirm the causal role of the target SNPs and explain the underlying pathways and mechanisms how the genetic factors affect the observed phenotypes [38,39]. Such multi-level knowledge may help to make clinical decisions in the future and create new cardiovascular disease therapeutics.

The limitation of our study is that we used a relatively small group of patients with CAD and a low death rate, however it is worth emphasizing that the study group is ethnically homogeneous and comes from the same region of residence (Upper Silesia), which minimizes errors resulting from heterogeneity, both ethnic and in terms of potential environmental factors. Another limitation may be also the fact that we did not use major adverse cardiac events (MACE) as end-point events, which is a common practice. Instead, we applied death from cardiovascular causes (CV death). In our opinion, CV death was the most important end-point regarding the aim and material analyzed in the study. We had no data on all CV events and this is why we could not analyze MACE as the end-point. Another limitation is that we applied *p* value < 0.05 as the threshold for genetic association significance, while some authors suggest that such *p* value may yield false positive results [40] and for example in Genome-Wide Association Studies (GWAS), the threshold for genetic association significance is much more stringent at *p* < 5 × 10^−8^ [41]. However, we tried to avoid false positive results by using multivariate analysis. The observed relationships seem to be also justified by the results obtained in the source studies and literature data on the associations with many aspects of the atherosclerotic process and its consequences.

## 5. Conclusions

The results of our study suggest that genetic variants of the *AGT, ABCA1*, and *CYBA* genes may be responsible for the differentiated survival of patients with CAD in the Caucasian population, which may be due to their pleiotropic effect on the initiation, course, and progression of coronary artery disease.

## Figures and Tables

**Table 1:** Analyzed polymorphisms in patients with CAD from Upper Silesia (Poland).

Locus	Gene	rs	Common Name	Risk Allele	Association with CAD	Gene-Gene or Gene-Traditional Risk Factor Interaction Increasing CAD Risk ^a^	Ref. No
1q23.3	*USF1*	2516839		T	-	+	[5]
		3737787		T	-	-	[5]
1q42.2	*AGT*	699	M235T (A > G)	G	+	+	[6]
2q35	*CYP27A1*	4674344		T	-	-	[7]
3q24	*AGTR1*	5186	1166 A > C	C	-	+	[8]
7q21.3	*PON1*	662	Q192R	Q	+	+	[9]
		854560		T	+	+	[10]
8q12.1	*CYP7A1*	3808607	278 A/C	C	-	-	[11]
		7833904		A	+	-	[12]
		8192879		G	-	-	[11]
		10504255		G	-	-	[11]
		10957057		C	-	-	[11]
		11786580		C	- ^b^	+	[11]
9p21	intergenic	10757278		G	+	-	[13]
9q31.1	*ABCA1*	2230806	R219K (G > A)	A	-	+	[14]
16q13	*CETP*	247616		C	+	+	[15]
		708272		C	-	+	[15]
		1532624		C	+	+	[15]
16q24.2	*CYBA*	4673	214C > T, C242T	T	-	+	[16]
		1049255	* 24A > G, A640G	G	-	+	[17]
		7195830	* 49 A > G	G	+	-	[18]
		9932581	−930 A > G	G	+	+	[19]
		13306296	−536 C > T	C	-	-	[20]
		16966671	−852 C > G	C	-	+	[20]
		72811418	−675 A > T	T	-	+	[20]
17q23.3	*ACE*	1799752	I/D, A287bpAlu	D	+	+	[21,22]
19q13.32	*APOE*	7412 ε2 429358 ε4	Epsilon1/2/3/4	ε4	-	+	[23,24]
22q13.31	*PPARA*	4253778	G > C, intron 7	C	-	+	[14]

^a^ + statistically significant in source studies, —statistically not significant; ^b^ association with myocardial infarction. CAD—coronary artery disease; * 3′-untranslated region.

**Table 2:** Cardiovascular causes of death in 5-year and 10-year follow-up.

ICD-10 Code	Cause of Death	5-Year Follow-Up		10-Year Follow-Up	
		n	%	n	%
I20.8	Ischaemic heart diseases. Other forms of angina pectoris.	0	0.00	1	3.13
I21.0	Ischaemic heart diseases. Acute transmural myocardial infarction of anterior wall.	0	0.00	1	3.13
I21.9	Ischaemic heart diseases. Acute myocardial infarction, unspecified.	6	42.86	9	28.13
I22.9	Ischaemic heart diseases. Subsequent myocardial infarction of unspecified site.	2	14.29	3	9.38
I24.9	Ischaemic heart diseases. Acute ischaemic heart disease, unspecified.	0	0.00	1	3.13
I25.0	Ischaemic heart diseases. Atherosclerotic cardiovascular disease, so described.	1	7.14	1	3.13
I25.1	Ischaemic heart diseases. Atherosclerotic heart disease.	0	0.00	1	3.13
I25.5	Ischaemic heart diseases. Ischaemic cardiomyopathy.	0	0.00	2	6.25
I25.9	Ischaemic heart diseases. Chronic ischaemic heart disease, unspecified.	2	14.29	6	18.75
I46.9	Other forms of heart disease. Cardiac arrest, unspecified.	0	0.00	1	3.13
I50.1	Other forms of heart disease. Left ventricular failure.	1	7.14	1	3.13
I61.6	Cerebrovascular diseases. Intracerebral haemorrhage, multiple localized.	0	0.00	1	3.13
I63.5	Cerebrovascular diseases. Cerebral infarction due to unspecified occlusion or stenosis of cerebral arteries.	1	7.14	1	3.13
I63.9	Cerebrovascular diseases. Cerebral infarction, unspecified.	1	7.14	1	3.13
I70.2	Diseases of arteries, arterioles and capillaries. Atherosclerosis of arteries of extremities.	0	0.00	1	3.13
I70.9	Diseases of arteries, arterioles and capillaries. Generalized and unspecified atherosclerosis.	0	0.00	1	3.13
Σ		14	100.00	32	100.00

ICD-10—International Statistical Classification of Diseases and Related Health Problems.

**Table 3:** Baseline characteristics of CAD patients (at the day of initial examination).

Characteristics	5-Year Follow-Up				10-Year Follow-Up			
	Dead ^a^ (n = 14)	Alive ^b^ (n = 262)	OR (95%CI)	*p*	Dead ^a^ (n = 32)	Alive ^b^ (n = 244)	OR (95%CI)	*p*
Age (SD)	45.92 (4.55)	45.33 (6.56)	-	0.80	44.29 (5.53)	45.49 (6.59)	-	0.18
Male, n (%)	12 (85.71)	178 (67.93)	2.83 (0.62–12.94)	0.16	25 (78.13)	165 (67.62)	1.71 (0.71–4.12)	0.23
BMI (SD)	26.91 (4.94)	27.16 (4.20)	-	0.67	26.30 (4.32)	27.25 (4.22)	-	0.23
Cigarette smoking, n (%)	11 (78.57)	150 (57.25)	2.74 (0.75–10.04)	0.11	21 (65.63)	140 (57.38)	1.42 (0.66–3.07)	0.37
Hypertension, n (%)	5 (35.71)	143 (54.58)	0.46 (0.15–1.42)	0.17	17 (53.13)	131 (53.69)	0.98 (0.47–2.05)	0.95
Diabetes mellitus, n (%)	4 (28.57)	19 (7.25)	5.12 (1.47–17.86)	0.02 *	6 (18.75)	17 (6.97)	3.08 (1.17–8.51)	0.05
Hypercholesterolemia, n (%)	4 (28.57)	70 (26.72)	1.10 (0.33–3.61)	0.78	10 (31.25)	64 (26.23)	1.28 (0.57–2.85)	0.55
Hypertriglyceridemia, n (%)	3 (21.43)	24 (9.16)	2.70 (0.71–10.37)	0.13	4 (12.50)	23 (9.43)	1.37 (0.44–4.26)	0.58
Mixed hyperlipidemia, n (%)	5 (35.71)	101 (38.55)	0.89 (0.29–2.71)	0.83	11 (34.38)	95 (38.93)	0.82 (0.38–1.78)	0.62
Critical stenosis, n (%)	11 (78,57)	163 (62.21)	2.23 (0.61–8.17)	0.22	25 (78.16)	149 (61.07)	2.28 (0.95–5.47)	0.06
Multivessel disease, n (%)	5 (35.71)	47 (17.94)	2.54 (0.81–7.93)	0.10	8 (25.00)	44 (18.03)	1.51 (0.64–3.60)	0.34

^a^ deaths from cardiovascular causes; ^b^ contains deaths from causes other than cardiovascular (n = 2 in 5-year follow-up and n = 7 in 10-year follow-up); * statistically significant difference.

## Data Availability

Not applicable.
